# Comparative Analysis on Alignment-Based and Pretrained Feature Representations for the Identification of DNA-Binding Proteins

**DOI:** 10.1155/2022/5847242

**Published:** 2022-06-28

**Authors:** Die Chen, Hua Zhang, Zeqi Chen, Bo Xie, Ye Wang

**Affiliations:** School of Computer and Information Engineering, Zhejiang Gongshang University, Hangzhou, 310018 Zhejiang, China

## Abstract

The interaction between DNA and protein is vital for the development of a living body. Previous numerous studies on in silico identification of DNA-binding proteins (DBPs) usually include features extracted from the alignment-based (pseudo) position-specific scoring matrix (PSSM), leading to limited application due to its time-consuming generation. Few researchers have paid attention to the application of pretrained language models at the scale of evolution to the identification of DBPs. To this end, we present comprehensive insights into a comparison study on alignment-based PSSM and pretrained evolutionary scale modeling (ESM) representations in the field of DBP classification. The comparison is conducted by extracting information from PSSM and ESM representations using four unified averaging operations and by performing various feature selection (FS) methods. Experimental results demonstrate that the pretrained ESM representation outperforms the PSSM-derived features in a fair comparison perspective. The pretrained feature presentation deserves wide application to the area of in silico DBP identification as well as other function annotation issues. Finally, it is also confirmed that an ensemble scheme by aggregating various trained FS models can significantly improve the classification performance of DBPs.

## 1. Introduction

DNA-binding proteins (DBPs) can bind and interact with DNA molecules in organic tissues, involving various cellular processes such as DNA replication, DNA repair, modification, and transcription regulation. The interaction between DNA and protein is of great significance in the gene study and the development of a living body [1]. Early detection experiments of DNA-binding proteins mainly adopt filter binding assays [2], genetic analysis [3], chromatin immunoprecipitation with DNA microarrays [4], and X-ray [5]. These approaches enable providing a detailed picture of binding for accurate DBP identification; however, they are usually costly and time-consuming. To avoid this disadvantage, much research has focused on the development of efficient machine learning methods for the identification of DNA-binding proteins.

Accurate identification of DBPs using machine learning methods is tightly coupled with precise information extraction from protein structures and sequences that, respectively, correspond to structure-based modeling and sequence-based prediction. The former by extracting high-resolution structure information such as solvent accessibility, torsion angle, and contact map [6–8] can output predictions with higher performance than a sequence-based predictor, but its main drawback is limited to a relatively small number of available three-dimensional structures as well as annotated functions. In contrast, a sequence-based predictor by extracting only sequence features is much more suitable for modeling on large-scale datasets. Recently, Zhang et al. [9] designed sequence-level features composed of pseudo amino acid composition (PseAAC), pseudo position-specific scoring matrix (PsePSSM), PSSM-transition probability composition (PSSM-TPC), and so on. Zou et al. [10] utilized four types of features concerning the multiscale continuous and discontinuous descriptor (MCD) [11], normalized Moreau–Broto autocorrelation (NMBAC) [12], PSSM-AB [13], and PsePSSM [14]. Hu et al. [15] extracted features by calculating AAC, PsePSSM, PsePRSA, and PsePPDBS. The study presented by Zhang et al. [16] focused on four different features: reduced sequence and index vectors (RS), PseAACs, PSSM-auto cross covariance transform (PSSM-ACCT), and PSSM-discrete wavelet transform (PSSM-DWT). As a summary, information extraction for a sequence-based DBP predictor mainly includes features such as physicochemical properties [17, 18], (pseudo) AAC [19, 20], predicted secondary structure and solvent accessibility [18, 21], PSSM [21–23], and their various variations, which also comprise the majority of features adopted in our previous work [24]. Among these features, the PSSM, as a sequence alignment-based representation generated by PSI-BLAST [22], is the most representative one compared to other types of features. Whether the prediction method is structure-based or sequence-based, PSSM has been widely adopted in the DBP classification task due to its underlying evolutionary profile with excellent performance. Besides, it is also widely accepted as the dominant sequence representation in various areas of structural bioinformatics, including the predictions of secondary structures [25], solvent accessibility [26, 27], contact map [28, 29], disordered region [30], DNA-binding proteins [9, 24], and function sites [31], to name just a few.

However, one run of the PSI-BLAST program on a long protein sequence is becoming more and more time-consuming due to the increasing number of sequences in the NCBI NR database (nonredundant protein sequence database). This may greatly limit the application of a DBP predictor due to the ambitious information extraction procedure if the PSSM features are taken into account. In recent years, with the popularity of unsupervised pretrained language modeling in the field of natural language processing (NLP) [32], protein language modeling aiming at the scale of evolution has also emerged in the area of computational biology and bioinformatics, such as ProtTrans [33], MSA Transformer [34], and ESM-1b [35]. These pretrained language models (pLMs) trained across millions of protein sequences that span evolutionary diversity learn some of the grammar of life language as well as the structures and the functions of proteins [34, 35]. It is confirmed that the resulting pretrained representations encode information about secondary and tertiary structures that can be identified using linear projections [35]. Compared with traditional alignment-based evolution scaling models such as PSSM, the novel pretrained language modeling has led to great advances in predictions of the protein structure and contact map without multiple sequence alignments, thereby bypassing the expensive database searches [33, 34].

Little attention by researchers has been paid to the application of pLMs to the identification of DBPs. The aim of this work is to provide comprehensively a comparative analysis on the alignment-based and the pretrained sequence representations. We design four types of features by using different averaging operations in a unified scheme for PSSM and ESM-1b representations. Next, performances of the features concerning PSSM and ESM-1b representations are firstly validated on six main feature sets with no feature selection (NFS) and then explored by utilizing various feature selection methods in the light of importance-based feature ranking. The resulting feature subsets optimized by the previous feature selection stage are further reduced by performing wrapper-based feature selection using the recursive feature elimination (RFE) strategy. Finally, an ensemble is simply constructed via the combination of all optimized classification models obtained in different feature selection stages. As expected, experimental results show that the ESM-type features in general outperform the PSSM-type features. Additionally, the support vector machine with linear kernels (LinSVM) and logistic regression (LR) are the two best approaches among the importance-based feature ranking methods. The proposed ensemble model based on all classification models optimized in the feature selection stages significantly improves the prediction performance on the independent test set.

## 2. Materials and Methods

### 2.1. Benchmark Datasets

Following our previous work [24], DNA-binding protein (DBP) sequences for model training and feature selection were extracted from the Protein Data Bank (PDB) [36] by searching the mmCIF keyword “DNA binding protein.” The entire DBP set after removing the chains with a length of less than 50 and character of “X” was subsequently filtered with CD-HIT [37] at 25% sequence identity. It is further filtered using CD-HIT at 25% identity against the independent set PDB186 [24]. These steps resulted in a set of 808 DNA-binding protein sequences that share 25% identity both with each other and with the DBPs of the independent set PDB186. On the other hand, 808 non-DNA-binding proteins were randomly selected from the sequences that were deposited in PDB after January 2018 and filtered using CD-HIT with 25% identity against the independent set PDB186. Finally, a dataset, called PDB1616, is created including 808 DBPs and 808 non-DBPs, which share 25% identity both with each other and with the independent dataset PDB186.

This new set PDB1616 is used to fit classifiers and perform various feature selection methods. Nevertheless, the PDB186 dataset composed of 93 DBPs and 93 non-DBPs, which has been widely adopted as an independent set for blind tests by a number of research groups [1, 38–43], is also used in this work to evaluate various feature selection models and compare performance with other baseline methods concerning the prediction of DNA-binding proteins. The PDB IDs of PDB1616 and PDB186 are listed in supplementary Table S1 and Table S2^1^, respectively.

### 2.2. Feature Representations

To comprehensively investigate the feature representation for the identification of DNA-binding proteins, we focus on two representative unsupervised models, i.e., the position-specific scoring matrix based on multiple sequence alignment (MSA) and the sequence representation based on pretrained protein language models.

#### 2.2.1. Position-Specific Scoring Matrix

A position-specific scoring matrix (PSSM) is an *L* × 20 matrix,

(1) $$\begin{array}{c} \text{PSSM}=\left[\begin{array}{cccc} s_{1,1} & s_{1,2} & \cdots & s_{1,20}\\ s_{2,1} & s_{2,2} & \cdots & s_{2,20}\\ \cdots & \cdots & \cdots & \cdots \\ s_{L,1} & s_{L,2} & \cdots & s_{L,20} \end{array}\right], \end{array}$$

where *s*_*i*,*j*_ stands for the score of the residue *i* mutated as an amino acid type *j* (*j* = 1, 2, ⋯, 20) during an evolutionary process and *L* is the sequence length of a protein. In our experiments, the normalized form using the sigmoid function, i.e., sigmoid(PSSM), is finally adopted as the feature representation matrix for a protein. The PSSMs of all proteins are generated by performing the PSI-BLAST program [22] of blast-2.10.1+ with three iterations and an *E* value of 0.001 against Swiss-Prot and RefSeq (NCBI Reference Sequence Database) that were released in June 2020. These sequence databases Swiss-Prot and RefSeq result in two representation matrices, denoted as PSSMS and PSSMR, respectively.

#### 2.2.2. ESM-1b

Recently, there is a growing interest in developing self-supervised learning approaches for protein sequence representation, named as protein language modeling at the scale of evolution, attributed to the great success in the area of natural language understanding. Rives et al. [35] proposed evolutionary scale modeling (ESM) via self-supervised learning by training a deep contextual language model with the Transformer [44] structure based on 86 billion amino acids across 250 million protein sequences spanning evolutionary diversity. As a representative pretrained protein language model, the proposed ESM-1b by Rives et al. [35] contains information about biological properties in its representations and correlation between residues as an end-to-end model which can realize the prediction of the contact map without the inclusion of traditional features such as PSSM [35]. ESM-1b outperforms all tested single-sequence protein language models, including UniRep [45], TAPE [46], SeqVec [47], LSTM, and Transformer, across a range of structure prediction tasks. In addition, a specialized ESM version for the prediction of variant effects, called ESM-1v, enables the efficient zero-shot prediction of the functional effects of sequence variations.

As a comparison with PSSM, we extracted the residue-level sequence representation generated by the ESM-1b model for all protein sequences in the training set and the blind test set. The resulting sequence representation, named as ESM, is an *L* × 1280 matrix as follows:

(2) $$\begin{array}{c} \text{ESM}=\left[\begin{array}{cccc} e_{1,1} & e_{1,2} & \cdots & e_{1,1280}\\ e_{2,1} & e_{2,2} & \cdots & e_{2,1280}\\ \cdots & \cdots & \cdots & \cdots \\ e_{L,1} & e_{L,2} & \cdots & e_{L,1280} \end{array}\right], \end{array}$$

where a row vector (*e*_*i*,1_, *e*_*i*,2_, ⋯, *e*_*i*,1280_) in the ESM matrix means a contextual representation vector of the *i*th residue in the sequence.

### 2.3. Feature Extraction

The abovementioned representation matrices of a protein, i.e., PSSMS, PSSMR, and ESM, are all residue-level representations. However, it is required to further extract sequence-level feature representation in order to investigate the prediction problem of DNA-binding proteins. Given a residue-level representation *R* = (*r*_*ij*_) with the shape of *L* × *d*, where *r*_*ij*_ = sigmoid(*s*_*i*,*j*_) and *d* = 20 for PSSMS and PSSMR and *r*_*ij*_ = sigmoid(*e*_*i*,*j*_) and *d* = 1280 for ESM, the matrix *R* can be also denoted as *R* = {**R**_1_, **R**_2_, ⋯, **R**_*L*_}, where **R**_*i*_ ∈ *R*^*d*^ is the representation vector of the *i*th residue in a protein sequence. Note that there are different dimensions between PSSM-type (*d* = 20) and ESM-type (*d* = 1280) representation matrices. To compare the PSSM-type and ESM-type features in a unified framework, we just simply designed four categories of sequence-level feature representations using averaging operations, including average representations over all residues, *k*-separation residues, residues with specific amino acid types, and residue-residue correlations.

#### 2.3.1. Average Representation over All Residue-Level Feature Vectors

The average sequence-level representation over all residue-level feature vectors is defined as follows:

(3) $$\begin{array}{c} \text{Avg}R=\frac{1}{L}\overset{L}{\underset{i=1}{\sum }}R_{i}, \end{array}$$

which is named as PSSMS_Avg, PSSMR_Avg, and ESM_Avg when *R* is PSSMS, PSSMR, and ESM, respectively. This extraction results in *d* sequence-level features.

#### 2.3.2. Average Representation over *k*-Separation Residues

All residues in a sequence can be divided into multiple subsets composed of residues with a given *k*-separation sequence distance, where *k* = 2, 3. The average representation over *k*-separation residues is defined as the vector averaging on these subsets as follows:

(4) $$\begin{array}{c} \text{ Avg}R\_\text{Sep}\left(k,s\right)=\frac{1}{N_{k,s}}\overset{N_{k,s}-1}{\underset{i=0}{\sum }}R_{ki+s}, \end{array}$$

where *k* = 2, 3, *s* = 1, 2, ⋯, *k* is the start residue position in the computation, and *N*_*k*,*s*_ = [(*L* − *s* + *k*)/*k*] denotes the number of feature vectors in the vector subset {**R**_*s*_, **R**_*k*+*s*_, ⋯, **R**_*ki*+*s*,⋯_} of the representation matrix *R*. This extraction results in 5*d* sequence-level features.

#### 2.3.3. Average Representation over Twenty Types of Amino Acids

Similarly, the entire set of all residues can be divided into 20 subsets corresponding to twenty standard amino acid types. The average representation over residues with specific amino acid types is defined as the vector averaging on these subsets as follows:

(5) $$\begin{array}{c} \text{Avg}R\_\text{AA}\left(t\right)=\frac{1}{N_{t}}\underset{A_{i}=t}{\sum }R_{i}, \end{array}$$

where *A*_*i*_ is the amino acid type of the *i*th residue, *t* represents one type of the 20 standard amino acids, and *N*_*t*_ denotes the number of residues with amino acid type *t*. This extraction results in 20*d* sequence-level features.

#### 2.3.4. Average Representation over Residue-Residue Correlations

In previous studies concerning feature extraction from PSSM, the pseudo position-specific scoring matrix (PsePSSM) [48] that aims at obtaining sequence order information has been widely applied to many function prediction fields, such as human protein subcellular localization [49], prediction of drug-target interaction [50], and identification of membrane protein types [51], to name just a few. Following the computation in PsePSSM, we define the averaging operation over the residue-residue correlations as follows:

(6) $$\begin{array}{c} \text{Avg}R\_\text{Corr}\left(\varphi \right)=\frac{1}{L-\varphi }\overset{L-\varphi }{\underset{i=1}{\sum }}\left(R_{i}-R_{i+\varphi }\right)⨀\left(R_{i}-R_{i+\varphi }\right), \end{array}$$

where ⨀ represents a pointwise multiplication for two vectors and *φ* denotes the sequence distance which is manually set to compute the correlations between two residues. From this extraction step, the number of resulting features is 3*d* given *φ* = 1, 2, 3.

As shown in Table 1, there are totally 29*d* features by integrating four categories of feature sets, i.e., 580 features named as PSSMS_All and PSSMR_All, respectively, for PSSMS and PSSMR representations with *d* = 20 and 37120 features named as ESM_All for ESM representation with *d* = 1280.

### 2.4. Feature Selection and Classifiers

In a situation with a limited number of samples but a great quantity of features, the classifiers may face a large computational cost and poor classification performance. Feature selection can be an alternative solution to reduce the dimensionality of feature space by deleting redundant features and improve the classification performance. All of the feature sets mentioned above are further examined using feature selection methods, including filter, embedded, and wrapper approaches.

#### 2.4.1. Filter-Based Feature Selection Methods

A filter method measures the correlations between individual features and classification labels. No classifier algorithm is utilized in this filter-based feature rank, which usually needs a scoring function. We choose feature variance [52], chi-squared statistics (Chi2) [53], and maximum information coefficient (MIC) [54] as representative filter-based methods in this comparative study.

As a typical and simple filter method, feature importance can be measured based on its feature variance, where a low variance of a feature means a small difference in all feature values. Meanwhile, Chi2 can be utilized to test the independence between variables. Similarly, MIC, which is capable of measuring the linear or nonlinear relationship between features and labels, has better performance than mutual information (MI).

#### 2.4.2. Embedded Feature Selection Methods

The goal of embedded methods is to select those attributes that are of great significance to the predictor fitted by a machine learning model. The features are then sorted by the feature importance outputs obtained by the predictor, or irrelevant and indistinguishable features are deleted from the entire feature set due to the lack of sufficient contribution to the prediction. We choose logistic regression (LR), linear support vector machine (LinSVM), and random forest (RF) [55] as representative predictors to generate feature importance values. In LR and LinSVM models, the importance of features can be obtained through the coefficients of different features in the linear combination. Besides, the random forest calculates impurity-based feature importance.

A feature subset can be also achieved by fitting a linear model with an added regularization term. As a representative regularization scheme, the Lasso algorithm [56] using the L1 norm estimates sparse coefficients of features, which can effectively reduce the number of features upon which the given solution is dependent. Moreover, another regularization method using the L2 norm with the advantage of stability, called Ridge regression [56], usually selects all features. As an alternative, ElasticNet combines these two regularization methods using both the L1 and L2 norms. This combination allows for learning a sparse model which maintains few nonzero weights like Lasso and the regularization properties of Ridge.

#### 2.4.3. Wrapper-Based Feature Selection Methods

The goal of wrapper-based feature selection is to search for an optimized feature subset accompanied with the training procedure of a learning estimator. As a representative search strategy, the recursive feature elimination (RFE) method is to select a feature subset by recursively pruning the least important feature from the current feature set. This procedure is recursively repeated on the pruned set up to the desired number of features. In practice, we choose RFECV in the scikit-learn platform [57] to perform the RFE algorithm in a cross-validation way to find the optimal number of features.

#### 2.4.4. Classifiers

The performance of the feature representation is actually coupled with an estimator. As a comparative study on the feature representation issue for the identification of DNA-binding proteins, we just examine the performance of several traditional classifiers released in the scikit-learn platform [57], including Gaussian naïve Bayes (GNB), *K*-nearest neighbors (KNN) [58], decision tree (DT) [59], logistic regression (LR), support vector machine (SVM) [60], random forest (RF) [55], gradient boosting decision tree (GBDT) [61], and eXtreme Gradient Boosting (XGB) [62].

The support vector machine (SVM) is a binary classification model, aiming at finding the largest separation hyperplane of positive and negative samples. It implements nonlinear classification by using nonlinear kernels instead of linear kernels. A GNB classifier calculates the probability of a given instance (example) belonging to a certain class in terms of Bayes' theorem and “naïve” independence assumption of two features [58] obeying Gaussian distribution. KNN outputs the prediction of an instance by searching for *K*-nearest neighbors from the training set. As a generalized linear regression analysis model, the logistic regression (LR) also calculates the probabilities describing possible outcomes that are modeled using a logistic function. The decision tree (DT) is aimed at creating a model by learning simple decision rules inferred from the data features. The random forest (RF) is an ensemble of multiple decision trees built by bootstrap samples with replacement from the training set. GBDT is a generalization of boosting to arbitrary differentiable loss functions. XGBoost (XGB), as an alternative implementation of the GBDT algorithm, introduces regularization and shows superior performance in many problems in various applications of data mining.

### 2.5. Experimental Steps and Performance Evaluation

In this study, we design a unified procedure for the above benchmark datasets, aiming at comparing PSSM and ESM features in the identification of DNA-binding proteins. The overall experimental steps concerning the feature selection procedure are summarized as follows.

#### 2.5.1. Experimental Steps

**Step 1 (data preparation).**

Any sequence in the training set PDB1616 and the test set PDB186 is firstly represented as a feature vector according to one of the six feature sets, i.e., PSSMR_All, PSSMR_Avg, PSSMS_All, PSSMS_Avg, ESM_All, and ESM_Avg. Then, all values of each feature in the training set are transformed using MinMax normalization, resulting in the feature range from 0 to 1.

**Step 2 (cross-validation).**

We perform fivefold cross-validation based on the training set where some feature selection is examined. When a feature subset is selected, a classifier is trained on the entire training set and is then applied to predict the test set. The classification quality in the training or test stage is evaluated using accuracy (ACC), Matthew's Correlation Coefficient (MCC), sensitivity (SN), and specificity (SP).

**Step 3 (feature selection procedure).**

After an investigation of the entire designed feature sets, we examine two stages of feature selection methods. The first stage is to select a number of top features based on feature importance ranked by several filter and embedded methods. Then, we further optimize the feature subset in the second stage by using a wrapper-based feature selection method, called recursive feature elimination with cross-validation (RFECV).

#### 2.5.2. Performance Evaluation

The performance metrics based on true positive (TP), true negative (TN), false positive (FP), and false negative (FN) are used to evaluate classification models. The following four metrics, i.e., accuracy (ACC), Matthew's Correlation Coefficient (MCC), sensitivity (SN), and specificity (SP), are included in this study.

(7) $$\begin{array}{c} \text{ACC}=\frac{\text{TN}+\text{TP}}{\text{TN}+\text{TP}+\text{FN}+\text{FP}},\\ \text{SN}=\frac{\text{TP}}{\text{TP}+\text{FN}},\\ \text{SP}=\frac{\text{TN}}{\text{TN}+\text{FP}},\\ \text{MCC}=\frac{\text{TN}\times \text{TP}-\text{FN}\times \text{FP}}{\sqrt{\left(\text{TP}+\text{FN}\right)\times \left(\text{TN}+\text{FP}\right)\times \left(\text{TP}+\text{FP}\right)\times \left(\text{TN}+\text{FN}\right)}}. \end{array}$$

## 3. Results and Discussion

### 3.1. Performance Comparison with No Feature Selection

Table 2 shows the performance comparison of eight baseline classifiers (GNB, KNN, DT, LR, SVM, RF, GBDT, and XGB) using six types of feature sets, including PSSMR_Avg, PSSMR_All, PSSMS_Avg, PSSMS_All, ESM_Avg, and ESM_All. Results for the training set PDB1616 are generated based on fivefold cross-validation (5CV). However, the prediction results on the test set PDB186 are reported by applying the classification models fitted over the entire training set. In addition, we just utilize default parameters for all baseline classifiers. As shown in Table 2, it can be observed that the support vector machine (SVM) with the Gaussian kernel has generally achieved the best prediction results in six feature sets when compared with the other seven baseline classifiers. In particular, the ACC measure of SVM is always the highest of cross-validation results over all six feature sets. Meanwhile, SVM almost performs the best test on PDB186 except in the case of PSSMS_All which is ranked the third.

Moreover, in regard to the aspect of features, the performance of the ESM representation is much better than those of PSSMR and PSSMS while the latter two PSSM representations are as expected close with a small ACC difference. For example, the performance scores of SVM in PSSMR_All and PSSMS_All are achieved by ACCs of 71.47% and 72.77%, respectively, while in ESM_All, the ACC value is 78.9% in the context of 5CV. In the case of predictions on the test set, ESM representation also shows significantly superior performance compared to PSSM representation. As a result, we can draw a general conclusion that ESM representation has a stronger identification ability of DNA-binding proteins than PSSM representation in the context of both the training set and the test set.

Finally, we also compare the discrepancy between the average representation of residue-level feature vectors and all designed features. Although there is a certain gap in the number of features between these two feature categories, we find that the gap is small, which is controlled at about 1%. This indicates that the average representation of all original residue-level feature vectors from ESM or PSSM in the context of cross-validation is comparable to all designed features. A small number of features used in the average representation can aid in reducing memory consumption and computational time in the training stage. This finding also serves to elucidate the necessity to perform feature selection on the entire feature set that has a large number of features, which is promising to reduce the computational cost and improve the prediction performance of DNA-binding proteins.

### 3.2. Feature Selection Based on Feature Importance

Due to the superiority of SVM with the Gaussian kernel that has been assessed on the training dataset, we choose SVM with the Gaussian kernel as the base classifier to train the classification models and carry out the comparison experiments of various feature selection methods. In addition, due to a small number of features, two feature sets (with only 20 features), PSSMR_Avg and PSSMS_Avg, are no longer considered in the present comparison experiments concerning feature selection.

**Figure 1:**
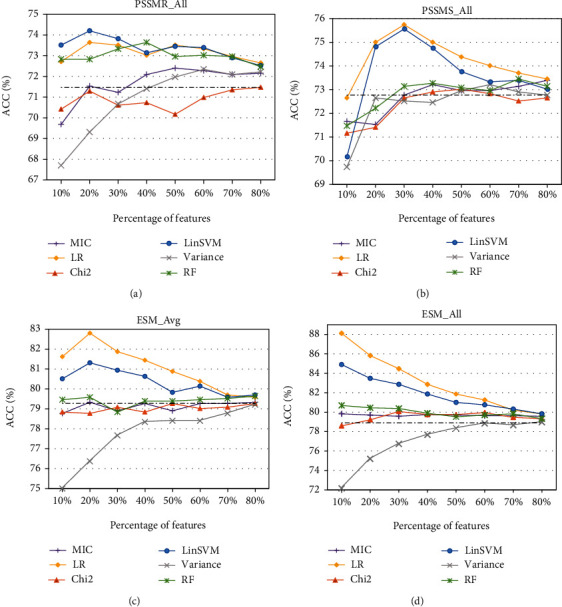
Result comparison of importance-based feature selection methods including MIC, Chi2, variance, LR, LinSVM, and RF that are investigated in the context of fivefold cross-validation based on four feature sets: (a) PSSMR_All, (b) PSSMS_All, (c) ESM_Avg, and (d) ESM_All.

Figure 1 shows the comparison of feature selection results based on feature importance ranked by three filter-based methods (variance, chi-squared statistics (Chi2), and maximum information coefficient (MIC)) and three embedded methods (logistic regression (LR), linear SVM (LinSVM), and random forest (RF)). To this end, firstly, all features in any set of PSSMR_All, PSSMS_All, ESM_Avg, and ESM_All are sorted by using the abovementioned six feature ranking methods. Then, feature subsets comprising from top 10% to top 80% with a step size of 10% in the sorted feature set are investigated and assessed by performing the SVM classifier with the Gaussian kernel in the training set. Figure 1 shows the plots of ACC values along with the top percentage of features in the four feature sets that have been sorted by the above six feature ranking methods.

**Figure 2:**
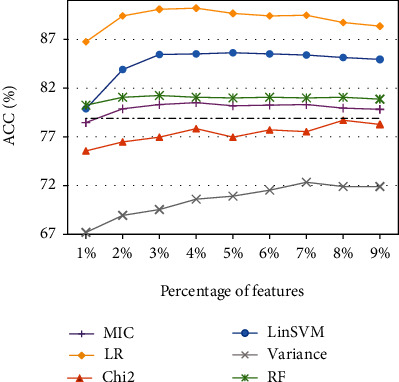
Plots of ACC scores of six feature ranking methods using less than 10% of features in ESM_All.

On the whole, with the increasing percentage of features, results of all feature selection methods gradually approach the performance achieved by the baseline task, i.e., the SVM with the Gaussian kernel using the entire feature set with no feature selection (NFS). It can be easily observed that the embedded feature ranking methods (LR, LinSVM, and RF) perform better than the filter-based methods (MIC, variance, and Chi2). Moreover, LR and LinSVM are the two best feature ranking methods, and LR performs relatively better than LinSVM in most cases. Finally, in cases of feature sets PSSMR_All, PSSMS_All, and ESM_Avg, the highest ACC values of LR and LinSVM are all achieved at 20% or 30% features, but the ACC curve of ESM_All just shows a decreasing trend with the highest ACC when 10% of features are selected. This implies that the ACC score may be boosted when the percentage of features is set to less than 10%. To achieve better results for ESM_All, we further investigate the percentage from 1% to 9% with a step size of 1% and perform the importance-based feature selection experiments. The ACC scores based on six feature ranking methods are shown in Figure 2.

As a result, in the case of ESM_All using less than 10% of features, LR and LinSVM still achieve the highest ACC scores as shown with an increasing trend from 1% of features and then a decreasing trend from 4% or 5% of features. To conclude, in four types of feature sets PSSMR_All, PSSMS_All, ESM_Avg, and ESM_All, the optimal results are achieved in cases of 20% features using LinSVM, 30% features using LR, 20% features using LR, and 4% features using LR, respectively. In addition, the remaining feature selection methods MIC, Chi2, and RF show similar performance, while the result of the variance selection is the worst even compared to the baseline task.

Overall, the results obtained by utilizing feature sets ESM_All and ESM_Avg are significantly better than those by PSSMR_All and PSSMS_All. In our opinion, it is primarily attributed to the fact that the number of features from the former representation is much more than that of the latter representation. To fairly compare the ESM-type and PSSM-type features, we further choose equal numbers of features from the above four feature sets to examine the performance of the current two optimal feature ranking methods LinSVM and LR.

**Figure 3:**
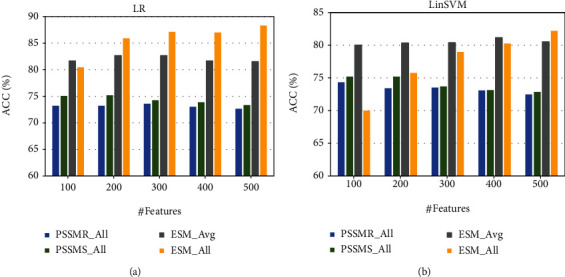
Performance comparisons using equal numbers of features for two optimal feature selection methods: (a) LR and (b) LinSVM.

Since the numbers of features in PSSMR_All and PSSMS_All are both 580, we investigate several cases using 100, 200,300, 400, and 500 top features for fair comparative experiments on the four feature sets. As shown in Figure 3, we can observe that most results of ESM representation are still significantly better than those of PSSM representation when the same number of features is used for model training. Regarding the feature sets PSSMR_All and PSSMS_All, LR and LinSVM show similar performance, while in ESM_Avg and ESM_All, the LR feature selection method is more superior and stable when compared with LinSVM. Therefore, it remains the same conclusion that ESM representation has superior performance than PSSM representation, which is again confirmed by a fair comparison using equal numbers of features.

Finally, we also performed three embedded feature selection methods using different regularizers based on the linear model, i.e., Lasso, LassoLars (Lasso model fitted with Least Angle Regression), and ElasticNet. These FS methods can directly result in feature subsets according to the sparse coefficients in the linear model. As shown in Table 3, the numbers of selected features by the above regularizers are all reduced a lot when compared with the numbers of features with no feature selection (NFS). In addition, it is again confirmed that ESM representation outperforms PSSM representation if the same FS is adopted. In the case of the largest feature set ESM_All, the classification performance based on 5CV achieves the best by the Lasso method in which 367 features are selected. Most likely, it is due to the large number of features designed in the ESM_All feature set. However, it is a little worse than the optimal result achieved by the importance-based feature ranking method LR using 4% features.

### 3.3. Results with Wrapper-Based Feature Selection Using the RFECV Method

To further refine the feature subset obtained in the previous stage using importance-based feature ranking, we perform the recursive feature elimination with cross-validation (RFECV), which belongs to wrapper-based feature selection methods. Only two feature ranking methods LinSVM and LR are included in RFECV experiments with regard to their excellent performance when compared with other feature ranking methods. The results are shown in Table 4, where “NFS” means no feature selection, “LinSVM*k*” and “LR*k*” (*k* = 30, 20, 5, 4) represent the optimized feature subsets gained by feature ranking methods LinSVM and LR using top *k*% features in the previous stage, respectively, and “LinSVM20_RFE” means the result obtained by carrying out wrapper-based feature selection RFECV started with the feature subset of LinSVM20. After feature selections using RFECV, the performance values such as ACC, MCC, SP, and SN on the training set are calculated based on fivefold cross-validation using the SVM classifier with the Gaussian kernel. The SVM model established on the entire training set PDB1616 is then applied to the predictions on the independent set PDB186. It can be discovered that the RFECV procedure just eliminates few features compared to the previous feature selection stage. In addition, the RFECV with LinSVM reduces a larger proportion of features than that using LR except in the case of ESM_Avg. Generally, the features eliminated by RFECV do not exceed 15% of the initial features. Two cases with the highest elimination ratios are deleted by 14.37% of features from the feature subset of LinSVM30 in PSSMS_All and by 14.82% of features from the feature subset of LinSVM5 in ESM_All.

As shown in Table 4, we find that the RFECV experiment can maintain the superior performance of the previous stage although several features after RFECV are eliminated. In particular, in the case of ESM_All, there is an improvement of ACC for both the 5CV and test results using the feature subsets from LR4 to LR4_RFE. However, the majority of ACC values in the second stage by performing RFECV decrease a little bit or remain the same as in the previous stage. Even in some cases, such as ESM_All, by changing the feature subset from LinSVM5 to LinSVM5_RFE, an improvement is achieved based on 5CV, but the test performance on PDB186 is decreased. We speculate that there may be a certain overfitting phenomenon without beneficial generalization by performing RFECV.

### 3.4. Ensemble Compared with Existing Predictors

We would like to stress that the goal of this work is to provide a comprehensive comparison between the alignment-based and pretrained feature representations for the identification of DBPs. Due to the simplicity of the averaging operations, we believe that the classification performance can be improved by designing more subtle features for PSSM and ESM representations. To improve the generalization performance, an alternative is the ensemble of the classification models established based on different FS methods. We simply propose an ensemble model, called the Feature Selection-based Ensemble model for the identification of DNA-Binding Proteins (FSEiDBP), by using a soft voting strategy. The proposed FSEiDBP integrates 34 classification models, including 32 models listed in Tables 3 and 4, plus two classification models constructed on entire feature sets PSSMR_Avg and PSSMS_Avg. The soft voting strategy is implemented by weighting ACC scores obtained on 5CV to the corresponding probability outputs of the classification models when a prediction is carried out. In detail, the output probability of a test sample belonging to a certain class *c* is calculated as follows:

(8) $$\begin{array}{c} p_{c}=\overset{34}{\underset{i=1}{\sum }}w_{i}p_{i,c}, \end{array}$$

where *w*_*i*_ = ACC_*i*_/(∑_*k*=1_^34^ACC_*k*_) and *p*_*i*,*c*_ ( *c* = 0, 1) is the predicted probability of the sample belonging to the class *c* for the *i*th predictor in the ensemble. In addition, ACC_*i*_ denotes the accuracy of the *i*th predictor evaluated on 5CV, *c* = 0 means non-DNA-binding protein, and *c* = 1 represents DNA-binding protein. As a result, the classification performance is significantly improved from the optimal result of FS experiments (achieved by LR4_RFE in the case of ESM_All) to the proposed FSEiDBP. In other words, as shown in Tables 4 and 5, the improvement is achieved by ACC values from 80.65% to 83.33% and MCC values from 0.6308 to 0.6733.

Table 5 also shows the performance comparison of independent tests obtained by the proposed FSEiDBP with 13 existing DBP predictors including DNA-Threader [63], DNAbinder [23], DNA-Prot [64], iDNA-Prot [65], DNABIND [66], Kmer1+ACC [67], iDNAPro-PseAAC [14], Wang's method [68], DBPPred [24], DPP-PseAAC [20], Local-DPP [69], iDBP-DEP [41], and iDNAProt-ES [70] where no pretrained feature representation is applied. The superior performance of PSEiDBP indicates that pLMs have stronger expression concerning feature information when compared with traditional feature representations. The performance improvement is also partly attributed to the ensemble of multiple models established on various FS experiments as well as entire feature sets with no feature selection.

## 4. Conclusion

Our work provides comprehensively interesting insights into the systematic comparison between the alignment-based and pretrained protein sequence feature representations at the scale of evolution for the identification of DBPs. The comparison analysis is firstly carried out with unified information extraction by applying several averaging operations to PSSM and ESM representations of protein sequences. This initial stage results in several feature sets concerning PSSM and ESM representations, which are compared by performing several classifiers aiming at the choice of the best machine learning method. The following comparison involves various feature selection experiments implemented with importance-based feature ranking methods and further recursive feature elimination based on the optimized feature subsets derived by the previous FS stage.

As expected, experimental results have confirmed that the pretrained ESM representation outperforms the PSSM-derived features in a fair comparison perspective using the same information extraction operations. Due to their simplicity, we are surely convinced of the performance improvement that can be achieved by designing much more delicate feature extraction operations on the PSSM and ESM representation matrices or integrating other types of features such as predicted secondary structure and solvent accessibility. To conclude, the pretrained feature presentation is recommended to be widely applied to the area of in silico function annotation for proteins. It is time to abstain from the time-consuming alignment-based evolutionary profile such as PSSM, especially in the prediction stage. In addition, our findings also include the suggestion on the choice of classifier and feature selection methods for the identification of DBPs. It is noticed that the proposed classification model of DBPs is not state-of-the-art. We would like to stress that our attention is mainly centralized on the comparison of alignment-based and pretrained feature representations. Designing much more delicate features or constructing an ensemble of multiple classification models in the light of different feature sets is most likely to improve the prediction performance. Therefore, an improved ensemble version of DBP predictors, called FSEiDBP, is established by integrating 34 models trained in FS experiments plus NFS experiments using a soft voting strategy.

## Figures and Tables

**Table 1:** Summary of features designed in this comparative study in which *d* = 20 for PSSMS and PSSMR representations and *d* = 1028 for ESM representation.

Index	Description about the feature category	Abbreviation	#Features
1	Average representation over all residue-level feature vectors	Avg*R*	*d*
2	Average representation over *k*-separation residues started with the *s*th residue	Avg*R*_Sep(*k*, *s*), *k* ∈ {2, 3}, *s* ∈ {1, 2, ⋯, *k*}	5*d*
3	Average representation over residues with a specific amino acid type *t*	Avg*R*_AA(*t*), *t* ∈ AA20	20*d*
4	Average representation over correlations between two residues given sequence distance *φ*	Avg*R*_Corr(*φ*), *φ* ∈ {1, 2, 3}	3*d*

**Table 2:** Performance comparison of baseline classifiers based on the fivefold CV and the test set using the entire feature sets.

Feature set (#Features)	Classifier	Fivefold cross-validation on PDB1616				Blind test on PDB186			
		ACC (%)	MCC	SP (%)	SN (%)	ACC (%)	MCC	SP (%)	SN (%)
PSSMR_Avg (20)	GNB	65.53	0.3108	67.08	63.99	61.83	0.2406	52.69	70.97
	KNN	66.09	0.3229	70.17	62.00	58.06	0.1613	56.99	59.14
	DT	60.89	0.2178	60.52	61.26	60.75	0.2157	56.99	64.52
	LR	69.74	0.3950	**71**.**16**	68.32	63.98	0.2856	53.76	74.19
	SVM	**70**.**85**	**0**.**4200**	64.98	**76**.**73**	**66**.**13**	**0**.**3395**	50.54	**81**.**72**
	RF	69.37	0.3875	68.32	70.42	65.59	0.3154	**58**.**06**	73.12
	GBDT	69.25	0.3849	69.06	69.43	65.05	0.3041	**58**.**06**	72.04
	XGB	68.19	0.3640	66.71	69.68	60.22	0.2047	56.99	63.44

PSSMS_Avg (20)	GNB	68.32	0.3666	66.34	70.30	68.28	0.3704	60.22	76.34
	KNN	68.13	0.3629	66.34	69.93	67.74	0.3552	65.59	69.89
	DT	62.93	0.2587	63.37	62.50	63.44	0.2689	62.37	64.52
	LR	70.92	0.4186	69.06	72.77	70.97	0.4242	63.44	78.49
	SVM	**73**.**21**	**0**.**4681**	66.71	**79**.**70**	**72**.**04**	**0**.**4563**	59.14	**84**.**95**
	RF	69.80	0.3962	68.19	71.41	**72**.**04**	0.4494	62.37	81.72
	GBDT	71.66	0.4336	**69**.**31**	74.01	**72**.**04**	0.4418	**68**.**82**	75.27
	XGB	69.06	0.3814	67.45	70.67	70.97	0.4242	63.44	78.49

PSSMR_All (580)	GNB	64.85	0.2975	67.70	62.00	60.75	0.2154	63.44	58.06
	KNN	59.34	0.2078	**81**.**19**	37.50	59.14	0.2025	**80**.**65**	37.63
	DT	59.84	0.1968	59.28	60.40	57.53	0.1506	55.91	59.14
	LR	68.44	0.3692	70.67	66.21	**67**.**20**	0.3443	65.59	68.82
	SVM	**71**.**47**	**0**.**4306**	67.82	**75**.**12**	**67**.**20**	**0**.**3465**	61.29	**73**.**12**
	RF	70.17	0.4044	66.83	73.51	62.37	0.2511	53.76	70.97
	GBDT	70.73	0.4146	70.67	70.79	64.52	0.2920	59.14	69.89
	XGB	69.18	0.3837	68.69	69.68	65.05	0.3051	56.99	**73**.**12**

PSSMS_All (580)	GNB	64.98	0.3083	**76**.**86**	53.09	57.53	0.1610	**75**.**27**	39.78
	KNN	62.56	0.2610	76.11	49.01	64.52	0.2920	69.89	59.14
	DT	63.06	0.2612	64.23	61.88	54.30	0.0866	48.39	60.22
	LR	69.74	0.3949	68.81	70.67	69.35	0.3873	67.74	70.97
	SVM	**72**.**77**	**0**.**4597**	65.97	**79**.**58**	73.12	0.4734	62.37	**83**.**87**
	RF	71.60	0.4337	67.08	76.11	73.66	0.4812	64.52	82.80
	GBDT	70.30	0.4061	68.81	71.78	**75**.**27**	**0**.**5073**	70.97	79.57
	XGB	70.24	0.4056	66.96	73.51	69.89	0.4012	63.44	76.34

ESM_Avg (1280)	GNB	71.35	0.4275	73.89	68.81	70.97	0.4209	75.27	66.67
	KNN	74.63	0.4927	73.64	75.62	72.58	0.4548	66.67	78.49
	DT	63.00	0.2599	63.49	62.50	61.29	0.2266	56.99	65.59
	LR	78.22	0.5646	76.86	79.58	78.49	0.5765	70.97	86.02
	SVM	**79**.**27**	**0**.**5908**	72.52	**86**.**01**	**79**.**03**	**0**.**5906**	69.89	**88**.**17**
	RF	74.32	0.4864	**74**.**50**	74.13	75.27	0.5055	**76**.**34**	74.19
	GBDT	76.67	0.5339	**74**.**50**	78.84	74.73	0.4960	70.97	78.49
	XGB	75.43	0.5090	73.64	77.23	77.42	0.5495	74.19	80.65

ESM_All (37120)	GNB	65.78	0.3235	76.73	54.83	58.60	0.1811	**74**.**19**	43.01
	KNN	64.17	0.2972	**79**.**21**	49.13	66.13	0.3269	32.69	58.06
	DT	60.27	0.2056	62.38	58.17	56.45	0.1318	46.24	66.67
	LR	78.28	0.5658	76.86	79.70	77.96	0.5666	69.89	86.02
	SVM	**78**.**90**	**0**.**5843**	71.53	**86**.**26**	**79**.**57**	**0**.**6087**	67.74	**91**.**40**
	RF	72.59	0.4520	70.79	74.38	73.12	0.4641	68.82	77.42
	GBDT	77.72	0.5550	75.50	79.95	75.27	0.5083	69.89	80.65
	XGB	77.41	0.5487	75.37	79.46	75.27	0.5112	67.74	82.80

*Note*. The number highlighted in bold is the best result corresponding to one feature set. An underlined number represents the optimal result over all feature sets.

**Table 3:** Results of embedded FS methods using three regularizers of the linear model in the light of 5CV on PDB1616.

Feature set	FS method	#Features	Fivefold cross-validation on PDB1616			
			ACC (%)	MCC	SP (%)	SN (%)
PSSMR_All	NFS	580	71.47	0.4306	67.82	75.12
	ElasticNet	188	72.77	0.4563	69.68	75.87
	Lasso	61	72.83	0.4577	69.43	76.24
	LassoLars	58	73.51	0.4707	71.53	75.50

PSSMS_All	NFS	580	72.77	0.4597	65.97	79.58
	ElasticNet	207	74.01	0.4847	67.20	80.82
	Lasso	54	73.82	0.4794	68.32	79.33
	LassoLars	38	72.77	0.4586	66.96	78.59

ESM_Avg	NFS	1280	79.27	0.5908	72.52	86.01
	ElasticNet	430	81.93	0.6442	75.37	88.49
	Lasso	142	83.11	0.6656	77.97	88.24
	LassoLars	151	82.43	0.6514	77.72	87.13

ESM_All	NFS	37120	78.90	0.5843	71.53	86.26
	ElasticNet	884	86.14	0.7267	80.94	91.34
	Lasso	367	**87**.**87**	**0**.**7598**	**83**.**91**	**91**.**83**
	LassoLars	250	86.70	0.7353	83.66	89.73

**Table 4:** Results of RFECV feature selections.

Feature set	FS method	#Features	5CV on PDB1616				Test on PDB186			
			ACC	MCC	SP	SN	ACC	MCC	SP	SN
PSSMR_All	NFS	580	71.47	43.06	67.82	75.12	67.20	34.65	61.29	73.12
	LinSVM20	116	**74**.**20**	**48**.**44**	71.91	**76**.**49**	**68**.**82**	**37**.**95**	62.37	**75**.**27**
	LinSVM20_RFE	110	**74**.**20**	48.43	**72**.**15**	76.24	68.28	36.73	**63**.**44**	73.12
	LR20	116	73.64	47.35	70.92	76.36	65.59	31.45	59.14	72.04
	LR20_RFE	114	73.58	47.22	70.92	76.24	66.13	32.58	59.14	73.12

PSSMS_All	NFS	580	72.77	45.97	65.97	79.58	73.12	47.34	62.37	**83**.**87**
	LinSVM30	174	75.56	51.36	**70**.**67**	80.45	73.66	48.12	64.52	82.80
	LinSVM30_RFE	149	75.00	50.34	69.18	80.82	71.51	43.94	61.29	81.72
	LR30	174	**75**.**74**	51.93	69.18	82.30	**74**.**73**	**50**.**31**	**65**.**59**	**83**.**87**
	LR30_RFE	157	**75**.**74**	**52**.**01**	68.69	**82**.**80**	73.66	48.33	63.44	**83**.**87**

ESM_Avg	NFS	1280	79.27	59.08	72.52	86.01	79.03	59.06	69.89	88.17
	LinSVM20	256	81.31	62.92	76.49	86.14	78.49	57.85	69.89	87.10
	LinSVM20_RFE	253	81.06	62.38	76.61	85.52	78.49	57.85	69.89	87.10
	LR20	256	**82**.**80**	**66**.**06**	**76**.**86**	**88**.**74**	78.49	57.65	**70**.**97**	86.02
	LR20_RFE	243	82.49	65.45	76.49	88.49	**79**.**57**	**60**.**28**	69.89	**89**.**25**

ESM_All	NFS	37120	78.90	58.43	71.53	86.26	79.57	60.87	67.74	91.40
	LinSVM5	1856	85.64	71.86	79.33	91.96	79.03	60.68	64.52	**93**.**55**
	LinSVM5_RFE	1581	87.07	74.61	81.44	92.70	78.49	59.36	64.52	92.47
	LR4	1485	90.22	80.75	85.89	94.55	80.11	61.81	**68**.**82**	91.40
	LR4_RFE	1392	**90**.**66**	**81**.**61**	**86**.**39**	**94**.**93**	**80**.**65**	**63**.**08**	**68**.**82**	92.47

*Note*. The number highlighted in bold is the best result corresponding to one feature set. An underlined number represents the optimal result over all feature sets.

**Table 5:** Performance comparison of the proposed ensemble FSEiDBP with other predictors validated on the independent dataset PDB186.

Methods	ACC (%)	MCC	SP (%)	SN (%)
DNA-Threader [63]	59.70	0.2790	**95**.**70**	23.70
DNAbinder [23]	60.80	0.2160	64.50	57.00
DNA-Prot [64]	61.80	0.2400	53.80	69.90
iDNA-Prot [65]	67.20	0.3440	66.70	67.70
DNABIND [66]	67.70	0.3550	68.80	66.70
Kmer1+ACC [67]	71.00	0.4310	59.10	82.80
iDNAPro-PseAAC [14]	71.50	0.4420	60.2	82.8
Wang's method [68]	76.30	0.5570	60.20	92.50
DBPPred [24]	76.90	0.5380	74.20	79.60
DPP-PseAAC [20]	77.40	0.5500	70.90	83.00
Local-DPP [69]	79.00	0.6250	65.60	92.50
iDBP-DEP [41]	80.10	0.6250	66.70	**93**.**60**
iDNAProt-ES [70]	80.64	0.6130	80.00	81.30
**FSEiDBP**	**83**.**33**	**0**.**6733**	76.34	90.32

## Data Availability

The data used to support the findings of this study are available within the manuscript and the supplementary file.

## Supplementary Materials

Supplementary Materials The file of supplemental data lists the PDB IDs of two datasets PDB1616 and PDB186.
