# Supplementary material for: Comparative Analysis on Alignment-Based and Pretrained Feature Representations for the Identification of DNA-Binding Proteins
Source: Comput Math Methods Med. 2022 Jun 28;2022:5847242. doi: 10.1155/2022/5847242 (PMC9256349; doi:10.1155/2022/5847242)
Supplement: Supplementary Materials — The file of supplemental data lists the PDB IDs of two datasets PDB1616 and PDB186. [file 5847242.f1.docx]

Supplementary material for ‘Comparative analysis on alignment-based and pretrained feature representations for identification of DNA-binding proteins’

**Table S1**. The list of PDB IDs for the training dataset PDB1616.

| **Type** | **PDB IDs** |
| --- | --- |
| DBP | 1U2WA,6CKNA,5W4UA,5BNVC,1BM9A,2KI2A,5W4UK,1F2RC,3LHKA,1VCCA,2KWQA,2AQLA,5HEKA,5UXXA,6E8CA,2PG4A,5W4UH,3MUJA,1VJFA,3P83D,3IVPA,4MUOA,6C5DA,6PY8B,6J07A,4RGLA,2KKVA,2NCJA,5YIXA,2L93A,1IYMA,3PGGA,5KYAA,1Z3IX,4QPJA,2C2JA,6IWVA,2L49A,5Z23C,5Z2VA,2RVBA,3TEDA,2A1JB,1DMLA,6E8EA,5ZTJA,4OWTB,2MDGA,5ZOKA,1Q87A,1K99A,5ZOJD,4FJOA,4JW3A,6EHIA,2VF7A,5TGQA,4GNXC,1U9NA,5EJLA,1NZPA,1WH5A,5A4NA,6OXBA,2DA6A,1WI3A,5D50A,5X3GA,6PY8F,4K2JA,1IFYA,2A1IA,1IXCA,5FGPA,3QVGB,5WTUA,6DT4A,2AN7A,2M14A,1UKLC,5WHGA,2D9HA,3K8AA,5JRBA,4A0KA,1C1KA,6OQMA,2O99A,4O8BA,5DOFA,3FDQA,4DHXB,5DYMA,3FYMA,3NCTA,6PLCA,6QJ5A,5ZADA,1T0FA,3QVGA,5G4VC,2JD3A,4HW0A,5NZWA,4KPYA,3SSCA,1JEQA,1Z1BA,2FMYA,3KHKA,4LVIA,2N00A,1CI4A,1SD4A,6BZFB,2N39A,1ZS3A,6VCSA,5UJ7C,1OV9A,2BA3A,1UL1X,6GUUA,5YDWA,2JTMA,5H45A,5UNKA,6QEQA,4Q47A,5DOKA,3BRFA,5VFXA,4E2IA,3ZQPA,2E6RA,2OZEA,1F2RI,2RRDA,6CKOC,5JR9A,1OSVA,1V63A,5I41B,6AILA,4XGCD,2LUAA,4RS7A,1SFUA,2RH3A,5YBXA,2ATQB,1OQJA,5FMPA,6P0FA,1X51A,6P7EE,2KHQA,2FWRA,5WC9A,5ZDZB,3ODCA,2GFUA,1TNSA,3P7NA,3GN5A,2LLHA,2QLCA,2P2RA,1V06A,1BW5A,3BTPB,4JW3C,3PGEA,2KW3A,3U21A,1KW4A,2NASA,5NR5A,5ODJA,5YIUA,5VA6A,6A57A,6J08A,6JQ1A,1WJ2A,1ZZKA,4FCYA,5KDMD,5U1JA,1C20A,3F6CA,4MTDA,5WX9A,2HGVA,1WG2A,6IF4A,5T7XA,1UOLA,3O27A,1D8BA,6DWWA,5ZB8A,4DFCA,5X3RA,2IO4A,6JUIA,2K75A,6MGNA,2B0LA,1KKXA,4M32A,6KKSA,5GNJA,1UKLA,3FHWA,1YDXA,6BZFA,4NDFA,4XGCF,6NVZA,1MSZA,2HUEA,5F3WA,2Z4RA,2INGX,5XE7A,5ITRA,2ADLA,3ON0A,3V62C,2K5VA,2JULA,2QSHA,2O8BB,3GXQA,2MRNA,6UXFA,1WG6A,1DP3A,3BU8A,2YRQA,2L4AA,4WZWA,4XGCC,5Z68A,5ZB2A,5JK7D,3II2A,4R4LA,3F6NA,2ZTCA,1ZP7A,2PI2E,5B02A,5GPDA,6SBWA,5YRQA,2M9MA,2ROHA,2N3XA,5YTPA,3PVPA,1GDTA,4O62A,1V1QA,4OWTC,2ATQA,3SQIA,6DGDA,4N6RB,2P6RA,2WBTA,6LW3A,2WKCA,1SQ8A,3WRPA,2L3NA,1WIDA,3EUSA,1WPKA,1F1EA,3WTPB,2VHGA,2K6GA,2L1PA,1H1JS,5BPDA,5GHRB,1IG6A,6C28A,2O8BA,3EQXA,3UFDA,4LDUA,3BS3A,1P1AA,6HP3A,4RYKA,1XWRA,4NXJA,5FGOA,3W3WB,2N5NA,1IN4A,3C1DA,3EYIA,1U3EM,2PI2A,1IV6A,3THOA,4FJOC,1P4WA,5DOBA,5HS7A,1WJVA,4Z47A,1Q1VA,2G9WA,4H9SE,5DYRA,6PCOA,6EUTA,3P9AA,4N0UE,3OLCX,2K9IA,3MAJA,2Q79A,1UL4A,1O57A,5YURA,5W9QA,4Z2MB,4Y00A,4TRKA,1QZGA,2AY0A,3U5ZF,6FY5A,4N0UB,6TPRA,1P4EA,1NEQA,5B42A,4O66A,3ERED,3MU6A,5DA7B,2NMUA,3KOJA,6MDWA,5WX8A,4PSLA,4ZTUA,5ZYTA,2JX3A,1IUFA,1T0FC,4WJAA,5NW5A,3AQQA,2DINA,2L92A,5WJBA,3II6X,1ULYA,1PRUA,1C6VA,4ZMIA,5D16A,3VKEA,6OZXA,1GCBA,1I11A,1ADUA,4XZGA,6BTCA,6L0OA,4N0UD,4NQWA,5W4UC,6HLKA,4LFUA,6DCRA,4IJHA,4JJNK,2MKDA,1RH6A,6K0WA,1Z4HA,3DSHA,3EZ2A,4ZTUB,6F5BA,3OD8A,1KZYC,5VFZA,5NL9A,4NB5A,1PL5A,5FUGC,1HCRA,6FWRA,2JR1A,1X57A,2LSSA,1HLVA,3K75B,1YUAA,4RZLA,5VC8A,5JK7F,2KFSA,6KO7A,6KI3A,4QICB,5ITJA,1WIJA,1PGZA,5LCLB,2VQCA,4A0KB,6H2XA,4FB3A,6FUUA,1AE2A,2WP0A,6IQOA,3THOB,6BOQA,5Z50A,2AXLA,2KKOA,2H9UA,6NOOA,5G2EA,1ZAEA,3HOSA,4DAPA,3K2AA,6QIBA,1WEEA,6LDMA,3QMBA,5ZHZA,5DOBB,4BJXA,1URJA,1OY3D,6HQ1A,5XYND,4WCGA,6RPRB,5W4UF,6MC8A,5UBDA,5W33A,5YRXA,1UFIA,2D8MA,5XXPA,1C6VX,4QICA,6C48B,3N4PA,5KO9A,6FB0B,6N0SA,5XXEA,6RASI,6M10A,2V79A,6CQKA,2ECCA,5XG3C,5JDKA,6JIPA,5W4UE,1KU5A,2G7OA,1P92A,3NFHA,6AMKA,3KJOA,5H65B,6AEPA,3U5ZB,1UB4A,5W4UB,1JB7B,3NXCA,4NW3A,3JU0A,2NBUA,4ZMKA,6ES5B,3POVA,6IS9A,4Y7RA,2OD5A,3F1ZA,2M3AA,1UB4C,2DMPA,6SEHA,4CI1B,5NOCA,2E5RA,4XGCA,4OM2A,3K4XA,3GZ5A,2D7LA,6RY3A,2WBRA,6GO3A,4HTOA,2F2EA,4OWWA,1O7IA,1USTA,5MRGA,5W4UI,2ICPA,2BDEA,4OOIA,6J4FB,3ZTHA,5DXZA,6HN7B,1AKHB,2L8EA,5V3MC,2BNWA,6KBUA,6C33A,6FBCA,2JPCA,6GAUA,2CJJA,3C1YA,4UA1A,1PFSA,5ZDZA,5JK7C,2OBPA,3I54A,2Z3XA,5AF3A,4OAZA,5DCXA,1RI7A,2WP0C,3BOSA,1CMBA,6I59A,1X3CA,6GW7A,5W1CA,3CNBA,2JMPA,3V20A,6NVPA,3EI4B,3WE2A,6MJ1A,1NGNA,1L8YA,5ZB2B,4GNXA,6VMTA,2DLKA,6C48A,2LFHA,1JE5A,2M8EA,5XEIA,5H69A,6QVFA,1YD6A,3TZDA,5YEJA,1HKQA,3GVAA,6AEBB,5FGUA,3ASKA,4PQLA,4XGCE,3KDEC,2MW8A,4PUGA,6OZWA,5YCQA,5GPAA,1OY3B,5D50E,6B1QA,5W2LA,5LKMA,2Q2KA,6TOBA,1Z91A,2GMGA,1YUIA,5F54A,1YSEA,6EQTA,2PKHA,2YVAA,1QZQA,2AIFA,3GV6A,5YBBA,5YWSA,5ZG9A,2DGZA,5Y26B,6E16A,5UK7A,2COBA,2JK1A,2FC7A,1X6FA,5FFJA,6GXDA,5XBTA,2VL6A,2DIMA,1WEUA,1JJRA,5N1CA,5ZCWA,5J6XA,5W4UL,5DOIE,4UUZC,6P7EA,4DHXA,6MEWA,4X01A,5X07C,6SEHB,2M9HA,6P7RA,1Y6UA,3QRFM,1KU9A,4X6GA,6ERFB,5CC1A,2DIGA,5OS9A,2NOGA,4PARA,5YIXB,6LNDK,3VW4A,2EBIA,3M03A,6ASBC,3V72A,4P0YA,5AN6A,2HKVA,3LDAA,1RW2A,5L7BA,1XD7A,2FPHX,6JTZA,6ACVA,2DA7A,6DF5A,5X5LA,2KW3C,1L3AA,3FRQA,6G3XA,3F6WA,4NQWB,4P55A,1J2FA,4PSNA,4ER8A,2P2UA,3G1CA,2MH3A,4N0UA,5G3JA,2BYKA,5XG2A,1F6VA,5VABA,1XV9B,2KNGA,6A8MA,2HQLA,2KKPA,3OA6A,1YJMA,6QLBA,2KIWA,5XYNC,1EE8A,1XP8A,5W3GA,5H65A,1JB7A,4YIFA,6QVIA,3NR7A,2EWTA,1ZRJA,2RVBB,2MLGA,1E17A,2OWYA,2ELHA,3WTPA,3U5ZA,1IRZA,6SY4A,5OMFA,6MRJA,3QO2A,1S6MA,3WPFA,3W3WA,1XCBA,5GHRA,2OKFA,2MAMA,4LQEA,3HI2B,1PVEA,3DPJA,1LRPA,6A44A,6DNWA,6HC3A,1OISA,5EYBA,3AL2A,1RMDA,2QUQA,2A1JA,3D6WA,6OGKA,5FCLA,1SFEA,4IX7A,2MH2A,2DQBA,2VS8A,5W4UJ,4Q77A,3NO7A,5UBFA,2KEBA,1RIFA,6KDVA,5XAYA,1N1JB,4YV6A,4O42A,1U78A,4DRBC,4LG8A,1T23A,2LFBA,2H6BA,4DWPA,5Y26A,1WEOA,6BUS1,5AKBC,2QSFX,6A7UA,4UMKA,2IJGX,5ZHCA,1FIAA,6JQSA,2ZDSA,5ZMMA,4RS8A,2W7OA,6J07B,5FMNA,2ZQEA,3IO5A,5ZUZA,1AKHA,5OVYA,6UVWA,6EZ1A,2EO0A,3ZEHA,4M6WA,6MGMA,4XGCB,2ALCA,6GVQB,6DA1A,3OKGA,6IUBA,4BJIA |
| nonDBP | 6HWEH,6K95A,6NS4A,6CDQA,6OUWA,6CWYC,5Z2HA,6UELA,6VOQA,5Z2PA,6T84A,6L18A,6M7XA,6SIUA,6OE9A,6H77Q,6CQCA,6AIGA,6EBOA,6IF6A,6E2HD,6EA3A,6H48A,6CI0A,6TTPB,6K9CA,6FSFA,6JG5A,6GNFA,6A0EA,6NWOA,6IE9A,6YV0A,6P8EA,6QGIA,6HBEA,6CWWA,6DDOA,6A9WA,6RS1A,6HHKA,6DYSA,6MRRA,6KQFD,6CITC,6CAFA,5ZGMA,6DD9A,6GGRA,6U44A,6FW2A,6PVGA,6CSJA,6QPSA,6O6JA,6QBEA,6QKGA,6UEWB,6P8PA,6QAZA,6VH5A,6HC6A,6IN7B,6MNIA,6DHVA,6JH0A,6LS5A,6AKQA,6I9NA,6HEEA,6C0YA,6U1RA,6EDDA,6FPLA,6C4VA,6CDKA,6QURA,6AGVA,6FMXA,6SFEA,6Y2DA,6G7DA,6K8ZA,6RYBC,6R6UA,6JYIA,6PNYA,6QRLA,6S2WA,6NBTA,6WZQA,6I17A,6U0IA,6CB2A,6I9AA,6CMVA,6IQTA,6O0WA,6DEFA,6W6DA,6ML2A,6G7WA,6JILA,6VUDA,6D4BA,6OD8A,6MJGA,6GZAA,6K06A,6OJBA,5ZZ6A,6JSBA,6EE2A,6PD2A,6CYZA,5ZW8A,6OK1A,6SPTA,6C25A,6P2IA,6NHFA,6MLKA,6O3WA,6HTFA,6R12A,6FMCA,6JPKA,6TEKA,6G0YA,6IX1A,6HPWA,6D96A,6G7BA,6PHVA,6MW7A,6QUCA,6O9YA,6H9FB,6TYYA,6OP8B,6AKWA,6OVIA,6NW9A,6MBXA,6O1TA,6QGTA,6CBXA,6CGOA,6ISCB,6JBRA,6KILA,6PX5X,6GOCA,6GQ0A,6GBSA,6H75A,6MQ5A,6FTTE,6L1YA,6NL2A,6IVVA,6UXTA,6K8TA,6N7OA,6EF6A,6OQQA,6S83A,6MS4A,6AAWA,6DGEA,6GPNA,6NFEA,6IU5A,6C29A,6I96A,6NVMA,5ZDAA,6PSMA,6MX5A,6VW8B,6N9GA,6WG4B,6MFAA,6PAKA,6HBZA,6DSPA,6CW3F,6MPXA,6KK8A,6EFGA,6ONPA,6JPLA,6HC8A,6FZTA,6IGGA,6VTJA,6N90A,6PXHA,6I0PA,6IHGA,6CXXA,6NPAA,6FXYA,6CUJA,6GTQA,6GGRB,6W03B,6FIYA,6HTFB,6U15A,6MHLA,6MBJA,6PGVA,6FWTA,6J0LA,6JFKA,6COMA,6GWKA,6ORTA,6FHPA,6DVHA,6KEAA,6Q63A,6OSPA,5QIVA,6S4QA,6ABWA,6H9FA,6H7FA,6SJ9A,6I5BA,6A71A,5ZS3A,6J2ZA,6L2DA,6V0MA,6J38A,6A48A,6UAKA,6T1TA,6HTKA,5ZW3A,6C9LA,6HPVA,6LJDC,6RFGA,6HCZA,6GV8A,6WI2B,5Z4TA,6C6NA,6A5KA,6QPLB,6NDUB,6IBGA,6UYHA,6QXDA,6HA7C,6R80A,6RGSA,6JHCA,6QXUA,6WAJA,6FPYA,6DBGA,6GVVA,6NALA,6K8VA,6INTA,6GU1A,6Y3PA,6S1UA,6DNQB,6O1XA,6IUFA,6R76A,6EB2A,6IA8A,6I5OA,6QBNA,6RB0A,5ZXDA,6EFKA,6MGCA,6V90A,6M9KA,6MELB,6D7YB,5ZXGA,6E0DA,6H5FA,6RZEA,6C30A,6A6JA,6JYVA,6QVOA,6GS2B,6SPNA,6H5XA,5Z8LA,6MPTA,6HWEJ,6KBRC,6CJNA,6DHUA,6E2AA,6O0AA,6ND7A,6N9HA,6RJCA,6K4EA,5QROA,6W4HA,6WQDB,6NIEA,6NW5A,6PGNA,6DEVA,6Q8UC,6M8NA,6I31A,6J2UA,6A5DA,6JO3A,6AH7A,6IWDB,6AAOA,6FZBA,6IX5A,6PYZA,6J7OA,6PREA,6U2AA,6FTOC,6J6OA,6OQAC,6U3WB,6CUPB,5QU5A,6QHGA,6XXYA,6P5TA,6J66A,6G9BB,6GUNA,6JLEA,6J4CA,6R44A,6H1ZA,6I1AA,6JTCA,6JCMA,6EDKA,6C3MA,6DX5A,6CW0A,6GMRB,6VMEE,6MJOC,6G3EA,6W9QA,6DHBA,6H24A,6GCBA,6QIDA,6RT8A,6ULXA,6J8MH,6QTAB,6JJSA,6KNYA,6Q8NA,6O7BB,6GU6B,6IDPA,6NU9A,6UUIX,5Z6BA,6I8WA,6JR7A,6U7TA,6GWJK,6FF0A,5QT1A,6H1WA,6CC7A,6UD7D,6UY1A,6UVPA,6LXSA,6I56A,6Y7FA,6AC5A,6GJEB,6SLKB,6NCHA,6J05A,6U3LA,6TMEC,6O31A,6N8QA,6CXUA,6KP5A,6NZXA,6FGJA,6MHDA,6A6SA,6VJBA,6A4TA,6NYVB,5Z6TA,6CWCA,6CPTA,6A7VD,6TKUA,6SC6A,6SDRB,6JQFA,6C4WA,6I50A,5ZFXA,6FU4A,6GZTA,6FTOA,6E1JA,5ZABA,6E8LA,6ST8A,6EE1A,6MV2A,6E60A,6E0LA,5Z6PA,6HLNB,6IW3A,5ZKTA,6II7A,6DS7A,6C7SA,6YIFB,6NY9B,6J13A,6QU6A,6ONOA,6J8MC,6N67A,5Z5EA,6PB3A,6GFIC,6CP8A,6A16A,6PIAA,6G49A,6R4HA,6HFMA,6SPVA,6IESA,6SCEA,5ZWZA,6MIUA,6V7GA,6L25A,6LPFA,6S22A,6ISPA,6GNUA,6KIAA,6A8UA,6EBNA,6DXOA,6ON1A,6GTAA,6FQ1A,6QIIG,6FOXA,6A7TB,5Z7BA,6NHIA,6GH8A,6O09A,6QO1A,5ZR8A,6NH9A,6NX0A,6MFUA,6CEYA,6W2WA,6HE0A,6JMUA,6CR0A,6KLRA,6QTKA,6JHMA,6QPKA,6USCA,6EGCA,6JNYA,6C7HA,6JOYA,6RV5A,6PHBE,6OX6B,6KQFA,6O6EE,6G55A,6GHDA,6T9YA,6H9DA,6J0QA,6POLA,6NAXA,6GYWA,6A6YA,6DF3L,6I3CA,6MWIA,6H2UA,6QB7A,6JMSA,6TZ3A,6A1KA,6DYZA,6LRGA,6RP3A,6OQ7A,6E85A,6OEWA,6CDWA,6TD9A,5ZJGB,6T5AE,6CXOA,6E0IA,6HWEI,6WOMA,6NJCA,6A5BA,6N54A,6D0PA,6IDCA,6M9GA,6PEDA,6TLKA,6JOWA,6XYNA,5Z3AA,6J0WA,6GJFA,6O0FA,6J8WA,6A07A,6P20A,6K17A,6TVPA,6MAVB,6CVKC,5ZMQB,6JELA,6QU3A,6GU2B,6GZJB,6CGMA,6LIVA,6W40A,6MICA,6AD3A,6H7DA,6RNQA,6ADZA,6QKMA,6PXAA,6IQ1A,5Z51A,6SU3A,6OWUA,6IJCA,6JW8A,5Z6ZA,6RZDA,6CBAA,6WGMA,5ZQNA,6HCDA,6OYCC,6DZ1A,6OVPA,6I2RA,6GH8B,6OZQA,6E87A,6VWWA,6JV7A,6G1IA,6DGSA,6P1GA,6MYSC,6HLBA,6FPIL,6OVTA,6G6JA,6FULA,5Z4GA,6WTTA,6MP7A,6HQ7A,6NI4A,6QKVA,6HS5A,6K2QA,6M92B,6R3SB,6OB3B,6V2DA,6VYEA,6JC0A,6GHOB,5ZFSA,6SU9A,6MK3A,6S5PA,6LEHA,6G1LA,6GFLA,6CZWB,6GRKA,6A6CA,6QV5A,6GBIA,6NPOA,6M9AA,6HIHA,6U7GC,6HKVA,6DI2A,6NIIA,6H6CA,6JFWA,6A8KA,6IMFA,6JL5A,6JNPB,6J34A,6C4QA,6LCQA,6UEKA,6D57A,5Z3RA,6MF4A,6QTKB,6W08A,6JZHA,5Z81A,6QROA,6DR7A,6SY9A,6HNMA,6W8BA,6A3AB,5Z5LA,6NXZA,6L1KA,6J0DA,6XWVE,6MBAA,5ZT3A,5Z38F,6QGTB,6JXNA,6CVOA,6G5AA,6C85A,6IRXA,6DQXA,6R9NA,6OAEA,6DA0A,6DEXA,6FOPA,6O4OA,6FTFB,6OHIA,6LQCA,6A3KA,5ZUMA,6NDTB,6EENA,6H96A,6OIEA,6NBOA,6LDQA,6EA6A,6FTQA,6JR1B,5ZX9A,6NDUA,6DGJA,5ZGNA,6CULA,6J36A,6T7OA,6OI4A,6CUQA,6JQ8A,6JAUB,6QJ0A,6HPRA,6S9EE,6I05A,7C23A,6IOYA,5ZTDA,6P9VA,6C7NA,6VW8A,6P20D,6VIJA,6IBUA,6E4NA,6NKFA,6RPYA,6IOVA,6GBOA,6CN8A,6TDGA,6H3XA,6RTPB,6OHMA,6IOWA,6L19A,6I9KA,6D0HA,6S9SA,6A34A,6G83A,6TD7A,6GWJD,6OK3A,6I2VA,6A73A,6UEWA,6UWOA,6DZGA,6KGIB,6S5QA,6EB0A,6GF6A,6IEOA,6SEAA,6AHWA,6JBXA,6N20A,6CHKA,6EEYA,6MFHA,6K09A,6DF3C,6HB5A,6G9PA,6THKA,6KLGA,6IU0A,6K3BB,6DG4A,6FSKA,6GE9A,5Z6DA,6CO6B,6J0YC,6MIWA,6QGWA,6A8WA,6JL0A,6H8OA,6FZZA,6FJEA,6RW2A,5ZOGA,6GPKA,6D7RA,6J56A,5ZT8A,6DHQA,6A9DA,6VDCA,6PZJA,6GH3A,6JPTA,6IBBA,6I3QA,6HF1A,6S8QJ,6HQJA,6A7VA,6O9AA,6MX1A,6FFVA,6JZJA,6RCKA,6P28A,6QFOA,5ZIBA,6PE3A,6NKLC,6C6BA,6SCFA,6C6GA,5ZIGA,6K09D |

**Table S2**. The list of PDB IDs for the independent test dataset PDB186.

| **Type** | **PDB IDs** |
| --- | --- |
| DBP | 2L8DA,2LI6A,2LJ6A,2LUYA,2LVSA,2LW1A,2LYHA,2LYJA,2MA1A,3AXJB,3B0CT,3Q8DA,3QODA,3QU3A,3R0JA,3R4KA,3RCOA,3RH2A,3RLOA,3S4WA,3S4WB,3S5RA,3SIAA,3TB6A,3TEKA,3TOCA,3TRBA,3TUOA,3U4VA,3U4ZA,3U50C,3ULJA,3ULXA,3UQZA,3UWXB,3UX8A,3V4GA,3V68A,3V9RA,3V9RB,3VH5A,3VH5W,3VIBA,3VK0A,3VPRA,3VZHA,3VZIA,3W03A,3W03C,3ZQJA,4A11A,4A11B,4ABXA,4ACOA,4AD8A,4ATHA,4BHXA,4BJ1A,4BLFA,4D8JA,4DAMA,4DG7A,4DKYA,4DRAE,4E1RA,4EOGA,4EQ6A,4EQ6B,4EXWA,4FE7A,4FW2A,4G12A,4G4KA,4G6DA,4G6DB,4GS3A,4H79A,4H7AA,4HD0A,4HIDA,4HLXA,4HPZA,4I1KA,4I99A,4I99C,4ICGC,4IDUA,4JLXA,4JOIA,4JOIC,4JOLA,4JQFA,4LC2A |
| nonDBP | 2LV4A,2LV9A,2LVFA,2LXUA,2LY4B,2LYVA,2LZEA,2M0YA,2YMBA,2YMVA,2YNQA,2YO3A,3J20A,3J21P,3J21R,3J21a,3J21k,3J21l,3J27A,3VYSC,3VYWA,3W1SC,3ZDWA,4AXNA,4AXQA,4AY9B,4AYFB,4AZ1A,4B0EA,4B29A,4B2XA,4B4TI,4B4TO,4B6AM,4B9JA,4B9KC,4BBKA,4FIPA,4FJ6A,4FL4C,4FLTA,4FM4A,4FM4B,4FMWA,4FMZA,4FNHA,4FQDA,4FY11,4FZQA,4G1EB,4G4SP,4G56B,4G59A,4G6XA,4G7HC,4G7LA,4G7XB,4G9JA,4G9QA,4GAKA,4GCWA,4GF2A,4GGDA,4GICA,4GIJA,4GIQA,4GNVA,4GOSA,4GVWA,4GWGA,4GWPF,4GWPG,4H3HB,4H5XA,4H63F,4HAMA,4HAOA,4HC6A,4HCEA,4HD1A,4HESA,4HGAB,4HN8A,4HN9A,4HPQC,4HQFA,4HVTA,4HWGA,4HWXA,4HY3A,4I1DA,4I8DA,4IAOC |
